# An Intracameral Injection of Antigen Induces *In Situ* Chemokines and Cytokines Required for the Generation of Circulating Immunoregulatory Monocytes

**DOI:** 10.1371/journal.pone.0043182

**Published:** 2012-08-17

**Authors:** Roshan Pais, Sourojit Bhowmick, Subhasis Chattopadhyay, Yen Lemire, Roshanak Sharafieh, Rajwahrdan Yadav, James O’Rourke, Robert E. Cone

**Affiliations:** Department of Immunology, University of Connecticut Health Center, Farmington, Connecticut, United States of America; University of Southern California, United States of America

## Abstract

Anterior Chamber-Associated Immune Deviation (ACAID) induced by an intracameral injection of antigen generates antigen-specific regulatory splenic T cells that suppress specifically cell-mediated immunity specific for the injected antigen. Circulating F4/80^+^ cells recovered from mice receiving an intracameral injection of antigen are thought to be ocular in origin and induce the development of thymic and splenic regulatory T cells. We have shown previously that after the intracameral injection of antigen there is a CCR2/CCL2-dependent infiltration of circulating F4/80^+^ cells into the anterior chamber associated with the generation of circulating, ACAID-inducing F4/80^+^ monocytes. Here we tested the hypothesis that the intracameral injection of antigen induces events in the anterior chamber that are associated with the induction of circulating immunoregulatory monocytes that induce the suppression of cell-mediated immunity. The intracameral injection of antigen resulted in aqueous humor (i) a time- dependent increase of CCL2 and CCL7, (ii) a transient increase in TNF-α, and (iii) an infiltration of CD11b^hi^, Gr1^hi^ and F4/80^+^ as well as F4/80^−^ and Gr1^hi^ peripheral blood cells into the anterior chamber. Further characterization of these F4/80^+^ cells revealed that they are Ly 6C^hi^, LY6G^lo^ or negative, 7/4 (LY6B)^hi^, CD115^+^, CD45^+^, CD49B^+^, and CD62 L^+^. Antibody-mediated neutralization of TGF-β *in situ* in the anterior chamber prevented the induction of circulating, ACAID-inducing monocytes and ACAID. These cells did not increase in the irides of ACAID-refractory CCR2–/– and CCL2–/– mice that received an intracameral injection of antigen. Our results extend our suggestion that ACAID is initiated as the result of a mild proinflammatory response to intracameral injection that results in the infiltration of a CCR2^+^ subset of monocytes into the anterior chamber where there is a TGF-β-dependent induction of an immunosuppressive phenotype in the infiltrated monocytes that recirculate to induce antigen-specific regulatory T cells.

## Introduction

The eye is an immune-privileged site that has unique anatomical features. Due to the lack of lymphatic drainage, aqueous humor in the anterior chamber is drained via the Canal of Schlemm/trabecular meshwork into the venous circulation. In addition to a lack of lymphatic drainage, tissues and fluids in the anterior and posterior chambers of the eye mitigate against immune/inflammatory reactions, thereby “protecting” sensitive ocular tissue from damage [1]. Moreover, the injection of antigen into the eye’s anterior chamber induces the antigen-specific suppression of cell-mediated immunity and the production of IgG2 antibodies to the same antigen as that injected into the anterior chamber. The suppression of delayed-type hypersensitivity (DTH) induced by the intracameral injection of antigen is effected by splenic CD8^+^ regulatory T cells specific for the injected antigen [1], [2]. Anterior chamber-Associated Immune Deviation (ACAID), well-demonstrated in rodents, has also been shown experimentally in non-human primates [1], [2]. Moreover, individuals with acute retinal necrosis display ACAID-like characteristics [3] suggesting that some ocular trauma could induce a systemic suppression of immune-based defense or pathology.

The intravenous transfer of murine F4/80^+^ monocytes recovered from the iris or circulation 24 hr after the intracameral injection of antigen (but not naïve F4/80^+^ cells) induces antigen-specific, splenic CD4 and CD8^+^ regulatory T cells that induce or effect respectively the suppression of DTH to the antigen injected into the anterior chamber [4]–[11]. These monocytes home to the thymus to activate regulatory thymocytes that in turn emigrate to the spleen. The monocytes also emigrate to the spleen where they interact with the recent thymic emigrants, antigen-specific CD4^+^ T cells and CD8^+^ T cells to induce CD8^+^ suppressor-effector T cells [2], [4], [7], [8], [10].

The exact origin of the circulating F4/80^+^ monocytes that induce regulatory T cells is under debate. Although the circulating, ACAID-inducing F4/80^+^ macrophages were thought to be derived from macrophages resident in the iris and ciliary body [1], [8], [9], the exit of such resident cells from the iris has not been demonstrated [12], [13]. However, recently we have shown that subsequent to the intracameral injection, there is an infiltration of circulating monocytes into the anterior chamber requiring the CCR2/CCL2 axis [5]. These monocytes recirculate to the thymus and spleen where they induce immunoregulatory T cells. Moreover, ACAID is not induced in either CCR2–/– nor CCL2–/– mice. Taken together, we proposed that the circulating monocytes that induce ACAID are recruited to the anterior chamber via the blood, and subsequently recirculate to the thymus and spleen [14]. Therefore, ACAID may be initiated in part as the result of a response to the intracameral injection itself. However, this response must be moderate because a florid inflammatory response in the anterior chamber could prevent the induction of ACAID [15].

After an intracameral injection, cells isolated from the iris have the capacity to induce ACAID when adoptively transferred to recipient mice. Moreover, *ex vivo* exposure of F4/80^+^ monocytes recovered from the peritoneal exudate to TGF-β in aqueous humor, a major contributor to the immunosuppressive environment of the anterior chamber [1], induces the ability of these cells to activate splenic suppressor T cells. Additionally, the intracameral injection of antibodies to Tumor Necrosis Factor-α (TNF-α) prevents the induction of ACAID [16]. In aggregate, these observations suggest that the monocytes that traverse the anterior chamber after the intracameral injection of antigen are induced to a “suppressive phenotype” by factors in aqueous humor as they recirculate through the anterior chamber. Yet, with the exception of the migration of circulating monocytes into the anterior chamber, the events that occur *in situ* in the anterior chamber shortly after the intracameral injection of antigen have not been described. Accordingly, we investigated *directly* the production of chemokines in aqueous humor induced by the intracameral injection of antigen, relationships between TNF-α in aqueous humor and the production of chemokines in aqueous humor that attract monocytes as well as the influence of TGF-β in aqueous humor *in situ* on the induction of ACAID.

Herein we report that the intracameral injection of antigen induces a rise in the aqueous humor of TNF-α and also the chemokines CCL2 and CCL7 essential for the infiltration of circulating inflammatory monocytes that express F4/80, CD11b and Gr1. ACAID does not occur unless these circulating cells are recruited to the anterior chamber. We suggest that the initiation of ACAID is based on factors of an immune-privileged environment that, through circulating cells generates a peripheral antigen-specific suppression of immune-mediated inflammation to a damaging stimulus. Further, ACAID is a “window” to determine the basis for the activation or activity of antigen-specific regulatory T cells that form an additional armamentarium of cell-mediated immunoregulation.

## Materials and Methods

### Mice

Female BALB/c, and C57Bl/6 mice 6–8 wks old were purchased from Charles River Laboratories,Jackson, ME., Harlan Laboratories, Frederick,MD or Jackson Laboratories, Bar Harbor, ME. CCR2–/– (B6.129S4-*Ccr2tm1Ifc*/J), CCR5–/– (B6.129P2-*Ccr5tm1Kuz*/J) MCP-1(CCL2)–/– (B6.129S4-*Ccl2tm1Rol*/J) mice were purchased from Jackson Laboratories. The mice were maintained in the Center for Laboratory Animal Care of the University of Connecticut Health Center. All work with animals was approved previously by the University of Connecticut Health Center Animal Care Committee (ACC-2007-369). All animals were treated according to the ARVO Statement for the Use of Animals in Ophthalmic and Vision Research.

### Injection of Antigen into the Anterior Chamber (AC, Intracameral Injection)

Naïve mice were anesthetized by intraperitoneal (ip) injection of ketamine (75 mg/kg)/xylazine (15 mg/kg). A 29 g needle was used to puncture the cornea and the aqueous humor was allowed to drain. The same needle attached to a cannula attached to a manually-controlled Hamilton syringe (Stoelting Co., Wood Dale, IL, USA) was inserted in the puncture and then 4–5 µl phosphate-buffered saline (PBS, pH 7.2) containing 50 µg ovalbumin (OVA, Sigma-Aldrich, Saint Louis, MO,USA)., was injected into the AC. The mice recovered ∼30 min after the injection; they exhibited no distress and began eating and drinking normally.

### Collection of Aqueous Humor and ELISA

Mice we euthanized by anesthesia with ketamine/xylazine followed by cervical dislocation. The mice were placed under a dissection microscope,aqueous humor was gently drained, and collected using an insulin syringe. Immunoassays of aqueous humor were performed as per manufacturers instruction for MCP-1 (CCL2) (eBiosciences, San Diego,CA USA), MCP-3 (CCL7) Antigenix, Huntington Station, NY, USA) and TNF-α (R&D systems, Minneapolis, MN,USA). A minimum of 8 biological sample wells were used per group in each assay. Concentrations of chemokines detected by ELISA were determined by a linear curve fit and assays were performed at least 3X.

### Intracameral Injection of TGF-α Blocking Reagents with Antigen

Mice received an intracameral injection of 4 µl of PBS containing 50 µg OVA, 3 µg monoclonal anti-TGF-β 1,2,3 IgG1 antibody (MAB #1835 R&D Systems, Minneapolis, MN, USA) or isotype control (MOPC 2 IgG (Sigma,St Louis,MO,USA), isotype control MOPC 21 IgG (Sigma, St Louis, MO, USA) antibody. TGF-β kinase inhibitor SB 431542 (#S431542 Sigma, St Louis, MO, USA) was dissolved initially in DMSO but subsequently diluted in PBS. The final intracameral injection contained 4 µl of 50 µg OVA and 10 µM SB 431542 in PBS. The vehicle control was done in a similar way but with DMSO only. A soluble TGF-β receptor II/Fc chimera (Cat # 523-R2, R&D systems MN), 150 ng in 4 µl of OVA-PBS was used for the intracameral injection with OVA. The same isotype (MOPC 21) was used in the control OVA-PBS intracameral injections.Wortmannin (#W1628-1MG, Sigma St Louis,MO,USA) was freshly dissolved in DMSO and further diluted in PBS. The final intracameral injection was 4 µl PBS/DMSO containing 50 µg OVA & 3–4 µM wortmannin. The vehicle control was done in a similar way but with only DMSO.

### Transfer of Peripheral Blood Monocytes Recovered from Mice that Received an Intracameral Injection of Antigen (AC- PBMCs)

Twenty-four hr after intracameral injection, blood was collected from the recipients of the intracameral injection. Blood was layered on Ficoll-paque (Stem Cell Technologies, Vancouver, Canada) and peripheral blood monocytes (PBMC) recovered from the whole blood as described [5], [8]. PBMC were counted using an Invitrogen (Grand Island, New York) Countess Automated Cell Counter and the recipient mice received 1×10^6^ cells iv AC- PBMCs from mice that received either isotype, control antibody or vehicle along with the antigen (OVA) served as positive AC-PBMC controls. AC- PBMCs from mice which received various forms of blockade of TGF-β or TGF-β activity along with the intracameral injection of OVA served as experimental AC-PBMCs. All the mice were immunized to OVA (please see below) 7 days after the injection of AC-PBMCs.

### ACAID Rescue

Immediately prior to the receipt of anti-TGF-β reagents, experimental group mice receiving reagents to neutralize TGF-β *in situ* were injected intravenously with AC- PBMCs recovered from naïve mice that had received an intracameral injection of OVA 24 hr previously.

### Immunization, Elicitation and Measurement of Delayed- Type Hypersensitivity (DTH)

Mice were sensitized by the subcutaneous (sc) injection of 200 µg ovalbumin (OVA) in 100 µl 1∶1 phosphate-buffered saline (PBS, pH 7.2) and Complete Freund’s Adjuvant (CFA, Sigma, St Louis, MO) 7 days after AC injection. DTH was measured 7 days after the mice were immunized. Sensitized or naïve mice were anesthetized with ketamine/xylazine (please see anterior chamber injection for dose) and footpad thickness was measured in triplicate with a digital engineer’s micrometer (Mitatoyo, Tokyo, Japan) before the footpads were challenged with antigen. OVA-sensitized or naïve mice received a 20 µl intradermal challenge injection of 100 µg OVA in PBS to one footpad and the thickness of the footpad receiving the challenge and the non- challenged footpad was measured 24 hr later. Micrometers of swelling were determined by computing the difference in thickness between the challenged and non- challenged footpad at time points before and after the challenge with OVA.

### Preparation of Iris Cells

Irides from at least 5 mice were recovered, incubated in collagenase/dispase, triturated and washed as described [5], [10] with some modifications. The digestion media as well as the subsequent wash media were prepared with a 5 mM HEPES Buffer and 5% heat- inactivated FBS in RPMI-1640. The cells were digested at 37°C for 10 minutes and then triturated with syringes containing needles with different gauges. The cells were then passed through a 70 µm cell strainer, washed twice in the wash medium and then used for flow cytometry.

### Flow Cytometry

Cells were incubated in staining buffer: PBS, 1% fetal bovine serum (FBS), 0.5 mM EDTA, 0.1% sodium azide with 0.5 mg/ml anti-CD16, CD32 blocking antibodies (Mouse BD Biosciences, Fc Block™ San Jose, CA) for 10 min at 4°C and then incubated for 30 min with 5–10 µl/1×10^6^ cells. Cells were also stained with color -matched rat isotype controls Antibodies used: Anti- F4/80 (Clone BM8), anti-CD11b (Clone 1/70), anti-GR1 (RB6-8C5), anti-CD115 (Clone AFS98), Anti- CD62L (Clone MEL-14) eBiosciences (San Diego,CA). Anti Ly-6C (Clone Al-21), Anti-Ly-6B.2 (Clone 7/4), Anti-CD49b (Clone DX5), AbD Serotec (Raleigh,NC).The cells were washed 3X with chilled PBS. The cells were analyzed by flow cytometry using a FACSCalibur Flow Cytometer and analyzed by FlowJo tristar A version7.8. A total of at least 10,000 cells were acquired for each sample.

### Statistics

Statistical significance for immunobiology experiments was calculated by one-way ANOVA. P-values were determined by the Student-Neuman-Keuls test.

## Results

### Intracameral Injection of Antigen Induces a Rise of CCL-2, CCL7 and TNF-α in the Aqueous Humor

**Figure 1:**
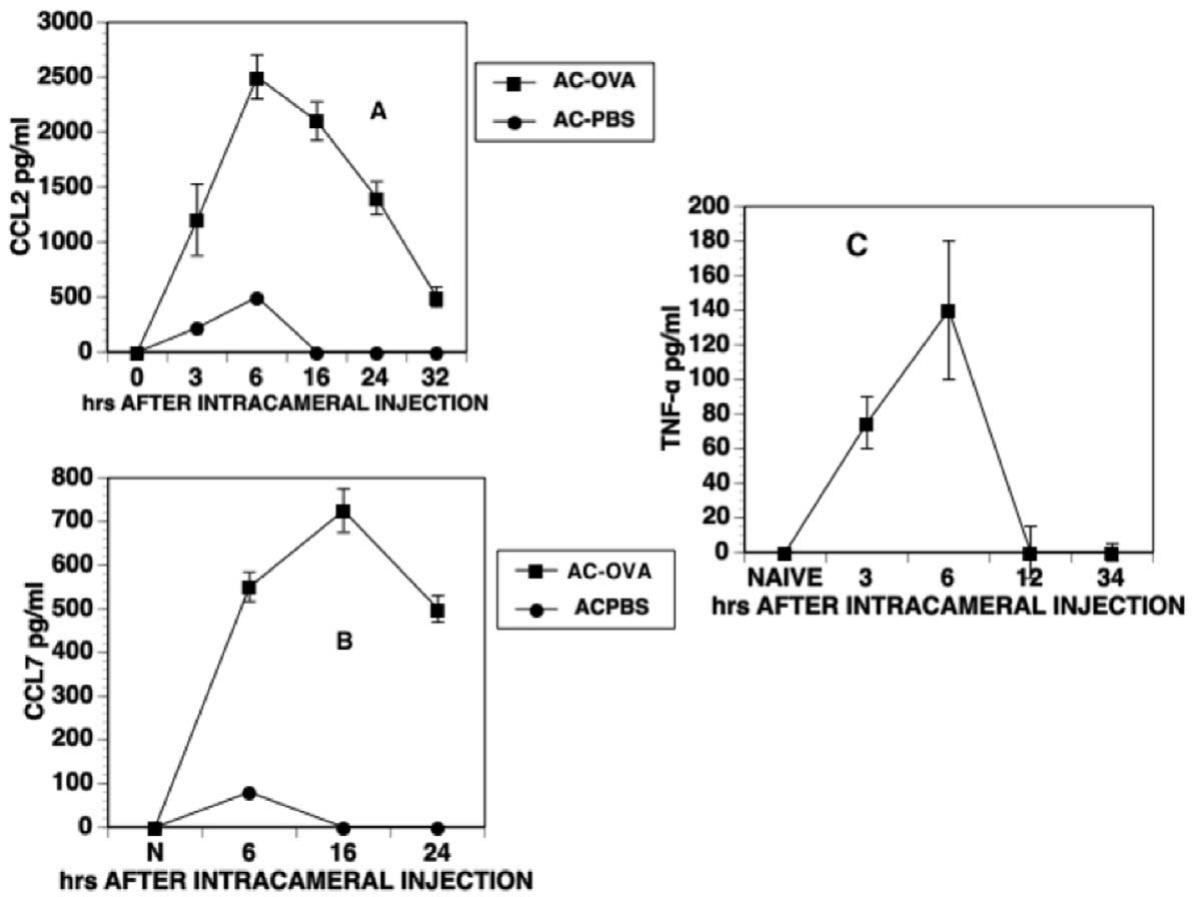
CCL2, CCL7 and TNF-α levels in the aqueous humor after intracameral injection. Aqueous humor was collected at different time intervals following the intracameral injection of OVA. CCL2, CCL7 and TNF-α levels in aqueous humor were detected by ELISA and are expressed as pg/ml. The data were pooled from 4 independent experiments for CCL2, 2 independent experiments for CCL7 and TNF-α and 2 independent experiments for MCP-3. A minimum of 6 replicates were used for each experiment.

The CCR2/CCL2 axis is essential for the infiltration of monocytes into the anterior chamber in response to an intracameral injection. Moreover, TNF-α is required for the induction of ACAID in response to the intracameral injection of antigen, [5], [16] and participates in a tolerogenic response to TGF-β-treated macrophages *in vitro* [17]. Accordingly, we investigated whether the an intracameral injection of OVA induced the production of TNF-α and the CCR-2 ligands CCL-2 and CCL-7 in aqueous humor. Naïve mice received an intracameral injection of OVA and aqueous humor was recovered at various time points after the intracameral injection. TNF-α, CCL-2 and CCL-7 levels in the recovered aqueous humor were determined by ELISA. Both CCL-2 and CCL-7 levels rose in aqueous humor 3–6 hr after the intracameral injection, peaked 16 hr post- injection and then declined (Fig. 1). A simple needle prick to the anterior chamber or the intracameral injection of PBS only caused a small and transient increase in either CCL-2 or CCL-7 of approximately 100–200 pg/ml 6 hr after injection (Fig. 1 A, B). However, this increase in CCL2 and CCL7 ceased 6 hr after the intracameral injection. Three-6 hr after the intracameral injection of antigen levels of TNF-α also rose from 0 pg/ml (naïve) and fell to 0 by 12 hr after injection (Fig. 1C).

### The F4/80^+^ Monocytes Recruited to the Anterior Chamber by CCL2 Induced by the Intracameral Injection of Antigen Express CD11b and Gr1

**Figure 2:**
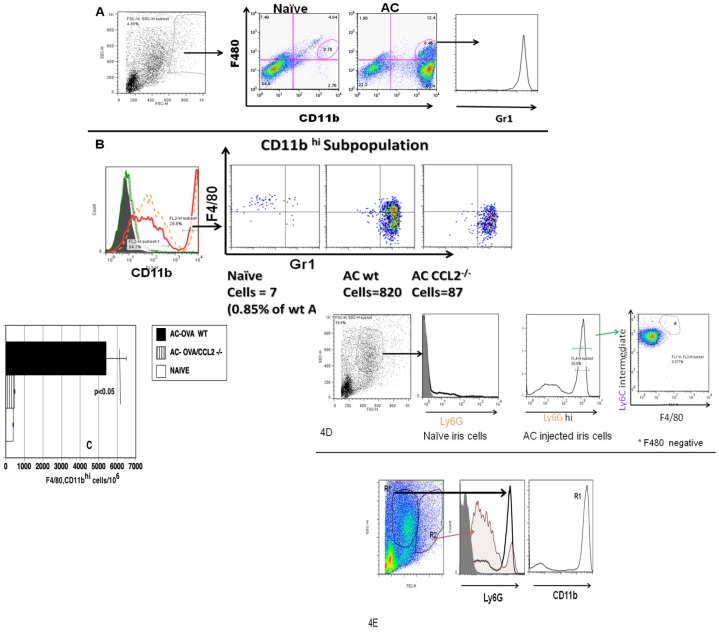
Intracameral (AC) injection results in infiltration of, F4/80^+^, CD11b^hi^ Gr1^hi^ monocytes and this process is greatly reduced in CCR2 or CCL2 null animals. Sixteen hours post intracameral injection of OVA irides were recovered from 10 eyes. A single cell suspension of the pooled irides was prepared and the cells were stained for specific monocyte markers as described in material and methods and compared the same with naive group or with cells from irides of either CCR2 null or CCL2 null animals treated identically. (A) Initial gating on the scatter plot is done as shown. A comparison between naïve iris monocytes and iris- gated monocytes recovered from mice receiving an intracameral injection of antigen (AC) stained with anti-CD11b, F4/80 and anti- Gr1. Only the group receiving an intracameral injection contains cells that are CD11b^hi^, F480^+^and Gr1^hi^. CD11b^hi^ but F4/80^−^ cells are also observed in mice receiving an intracameral injection of antigen. The figure is representative of 8 experiments (P<0.001). (B) The composite histogram shows that CD11b^hi^ population in AC groups but not in naïve group. The CD11b^hi^ F4/80^+^ Gr1^hi^ cells are increased in AC wild type group but only marginally increased in CCL2^−/−^ group after AC injection. (C) The bar diagrams shows the F/480^+^ cell population present in the wild type and CCL−/− mice receiving an intracameral injection as cells/10^6^ acquired. The figures are representative of 2 experiments. (D) AC injection results in neutrophil infiltration. Cells are gated on a population of higher SSC and intermediate FSC which in AC injection group show a Ly6G^hi^ peak. This population is absent in naive iris. Ly6G cells are F4/80 negative and Ly6C intermediate. (E) Comparison of 2 populations of cells in the AC injected iris group, based on their scatter properties. Ly6G hi cells are abundant in the R1 region but also are present in R2 region. These cells are also CD11b^hi^.

Because the induction of ACAID requires the CCR2-dependent infiltration of F4/80^+^ monocytes into the anterior chamber [5], the infiltrating monocytes might have the phenotype of a potentially inflammatory subset of monocytes [18], [19]. To test this hypothesis, mice received an intracameral injection of OVA. Irides were recovered 16 hr after the intracameral injection and the recovered cell suspensions stained with anti-F4/80 and anti- CD11b antibodies. The scatter properties of granulocytes, monocytes and lymphocytes allow them to be distinguished from each other and from cellular contaminants. Granulocytes including neutrophils typically have a high side scatter (SSC) and an intermediate forward scatter (FSC). The monocytes were gated around a relatively broad area of high forward scatter as they are larger than the lymphocyte population and low side scatter as monocytes are agranular. As shown in Fig. 2A, a CD11b^hi^ population of cells not present in the naïve irides was detected in irides recovered from mice receiving an intracameral injection. Among these cells are CD11b^hi^ cells that are F4/80^+^ (monocytes) and those that are F4/80^−^. These F4/80^+^, CD11b^hi^ cells also express Gr1^hi^. The F4/80^−^ cells were also found to express Gr1^hi^ and Ly6G^hi^ (data not shown, Figure 2D), suggesting that they may be neutrophils. A comparison of populations using gates based on their scatter properties show that Ly6G hi cells are abundant in a region of high side scatter and intermediate forward scatter but are also present within the broader monocyte gate which fell in the region of lower side scatter, a region occupied predominantly by monocyte/macrophages (fig. 2 D& E). Moreover, these F4/80^−^ cells are present only in the group receiving an intracameral injection but not in naïve eyes (Fig. 2D). Based on their abundance in a region typical for granulocytes (Hi SSC, intermediate FSC) and their CD11b^hi^, Gr1^hi^, Ly6G^hi^, Ly6C^intermediate^ and F4/80^−^ staining properties indicate that they are neutrophils [20], [21]. Since ACAID-inducing monocytes are F4/80^+^ [1], [6], [8], [10] and we have shown previously that ACAID is not induced in CCL2–/– mice, we reasoned that the production of CCL2 in the aqueous humor after an intracameral injection of antigen (Figure1) attracted these monocytes to the anterior chamber. Accordingly, to characterize these cells further as potentially ACAID-inducing circulating monocytes, single cell suspensions from the irides of naïve wild type, CCL2–/– mice, and wild type mice receiving an intracameral injection of OVA were stained for F4/80, CD11b and Gr1, a marker for cells of the CCR2 subset [18], [19]. A comparison of CD11b^hi^ cells for F480 vs Gr 1 between these groups (Fig. 2B and 2C) shows that in the wild type mice receiving an intracameral injection of OVA, the F4/80^+^, CD11b^hi^, Gr1^hi^ population is present prominently when compared to the naïve group.The increase of these F4/80^+^ CD11b^hi^ Gr1^hi^ cells is found to be marginal in the CCL2^–/–^ mice when compared to wild type mice receiving an intracameral injection of antigen suggesting that the increase of this population of cells in the anterior chamber is due to the production of CCL2.

**Figure 3:**
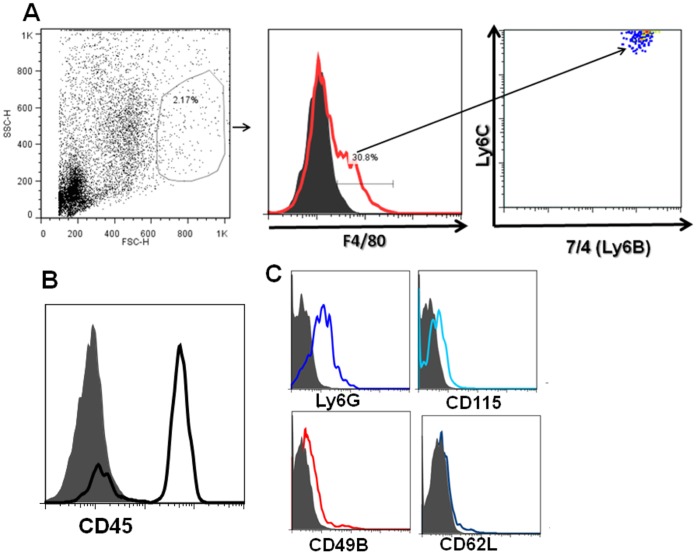
F4/80 and CD11b^+^ cells infiltrating the iris after the intracameral injection of antigen also express Ly6C, 7/4 (Ly 6B) and CD45. Preparations of naïve iris cells and cells recovered after an intracameral injection of antigen were stained with anti- F4/80 and (A) Ly6C and 7/4 (B) show a characteristic CD45 peak C) Ly6G**^lo^** or negative, CD115, CD49b^+^, CD62L**^lo^**. The figures are representative of 2 experiments. All the isotype controls in the histograms are shown as shaded.

Gr1 is a dimeric surface molecule composed of Ly6C and Ly6G and anti-Gr1 antibody (RB6-8C5) stains for both ly6C and Ly6G bearing cells [22]. Since CCR2^+^ monocytes are known to express higher levels of Ly6C [19], further analysis of this intracameral injection- associated population was performed as shown in fig. 3. Single cell suspensions from the irides recovered from wild type mice (C57/B6) receiving an intracameral injection of OVA were stained for F4/80 and markers within the F4/80^+^ population. As shown in Fig. 3A, these F4/80^+^ cells express Ly6C^ hi^ and 7/4(Ly6B). These cells express weakly Ly6G (Fig. 3 C), a marker expressed strongly in granulocytes (Fig. 2 D and E). In addition, these F/480^+^ cells also express CD45 and CD115 (fig. 3 B and C) and other minor markers thus indicating that these are indeed a CCR2^+^ subset of monocytes. Based on the observation that these cells show a Gr1 (Ly6C), CD11b^hi^ expression along with distinct CD45 expression and because these cells are greatly diminished in the irides of CCL2–/– mice after an intracameral injection of antigen, these cells are most likely to be freshly recruited from the highly vascular iris under the influence of CCL2 production in the aqueous humor induced by the intracameral injection.

### Regulation of the Increase in TNF-α and CCL2 in the Anterior Chamber after an Intracameral Injection

**Figure 4:**
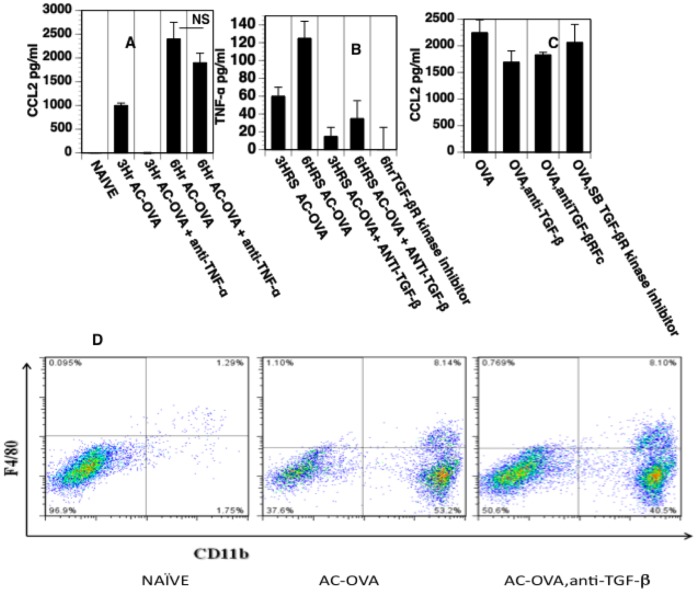
Regulation of the increase in TNF-α and CCL2 in the anterior chamber after an intracameral injection. Aqueous humor was collected at at 3 and 6 hour intervals following the intracameral injection of OVA. CCL2 and TNF-α levels in the aqueous humor were measured by ELISA. (A) Neutralization of TNF-α prevents the early rise of CCL2. Mice received an intracameral injection of OVA +/– anti-TNF-α antibody or isotype control. Neutralization of TNF-α via anti-TNF-α antibody at the time of AC injection significantly (P<0.05) reduced the early (3 hr) rise of (CCL2) MCP-1 but not at 6 hours. The data were pooled from 2 independent experiments and a minimum of 6 replicates were used in each group for each experiment. (B) Neutralization of TGF-β via a neutralizing antibody or TGF-β receptor kinase inhibitor (SB-431542) results in a significant reduction(P<0.05) in the rise of TNF-α induced by an intracameral injection of OVA. The data were pooled from 2 independent experiments. A minimum of 6 replicates were used for each experiment. (C) Neutralization of TGF-β via anti-TGFβ neutralizing antibody, Soluble TGF-β receptor or by TGF-β receptor kinase inhibitor (SB-431542) does not influence the CCL2 levels in the aqueous humor.(D) Neutralization of TGF-β by intracameral injection of anti-TGF-β with OVA does not block the infiltration of monocytes into the anterior chamber. Irides were removed 16 hr after intracameral injection and cell suspensions stained for Gr1 and CÎ11b. Experiments were repeated 3X.

The results presented above suggest that the intracameral injection of antigen induces an early, moderate response that potentially recruits circulating monocytes to the anterior chamber essential for the induction of ACAID. Because TNF-α is an essential element in the induction of ACAID [16] and participates in a “tolerogenic” response to TGF-β-treated macrophages *in vitro* [17], we investigated whether the production of TNF-α in the anterior chamber after intracameral injection is influenced by TGF-β in the anterior chamber. Moreover, to determine whether TNF-α or TGF-β in aqueous humor influence the production of CCL2 and the intracameral injection–induced infiltration of monocytes into the anterior chamber, anti-TNF-α or neutralizing anti-TGF-β 1,2,3 antibody or a TGF-β receptor-associated kinase inhibitor was included in the intracameral injection of OVA. Three and 6 hr after the intracameral injection, levels of CCL2 and TNF-α were measured in recovered aqueous humor. The infiltration of monocytes to the recovered irides were monitored at the 16 post intracameral injection and the same time point followed throughout. The intracameral injection of anti-TNF-α with OVA prevented an early increase in CCL2 in aqueous humor (3 hr) and reduced but did not eliminate a production of CCL2 six hr after the intracameral injection of OVA and anti-TNF-α (Fig. 4A). The increase in TNF-α in aqueous humor following the intracameral injection of antigen was markedly reduced or prevented by the inclusion of anti-TGF-β or an inhibitor of a TGF-β receptor-associated kinase in the intracameral injection (Fig. 4B). In agreement with this lack of inhibition of CCL2, the infiltration of monocytes was not affected by the blockade of TGF-β (Fig. 4C, Fig. 4D).

### Neutralization of TGF-β *in situ* in the Anterior Chamber Prevents the Induction of ACAID

**Figure 5:**
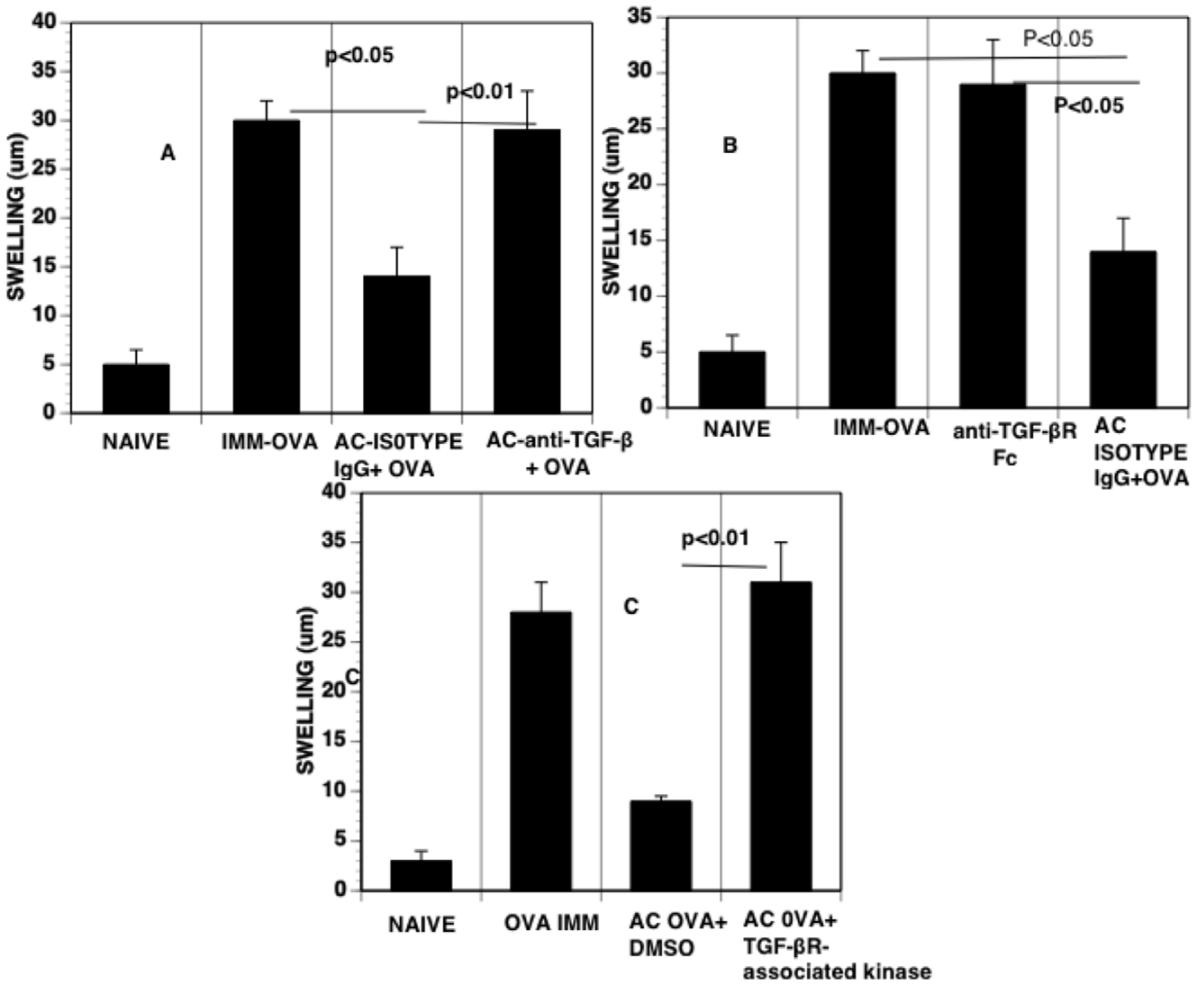
Blockade of TGF-β via (A) neutralizing antibody, (B) TGF-β soluble receptor, C) TGF-βreceptor kinase at the time of the intracameral injection of OVA on the development of DTH suppression. Intracameral injection of OVA included either vehicle only or isotype antibody control (AC positive control) or various TGF-β blocking treatments. The DTH measurements were performed 7 days after these groups were immunized with OVA. Each experiment, 5 mice/group, was conducted 3X.

TGF-β in aqueous humor is central to the induction of monocytes that induce ACAID [23]. However, the role of TGF-β *in situ* in aqueous humor for the induction of imunoregulatory monocytes has not been demonstrated directly. Accordingly, we neutralized TGF-β in aqueous humor *in situ* in the anterior chamber by including neutralizing antibody to TGF-β 1,2,3 (Fig. 5A), soluble TGF-β receptor (Figure 5B) or TGF-β receptor kinase inhibitor (Fig. 5C) with OVA injected into the anterior chamber to induce ACAID to OVA. After subsequent immunization, the footpads of these mice were challenged with OVA to determine the expression of DTH to OVA. Blockade of TGF-β by anti-TGF-β antibodies or a soluble receptor for TGF-β in the anterior chamber prevented the induction of the suppression of DTH to OVA induced by the intracameral injection of OVA. Neutralization of TGF-β however did not have any effect on CCL2 production or on the infiltration of F480 CD11b^hi^ positive cells induced by the intracameral injection (Figure 4).

**Figure 6:**
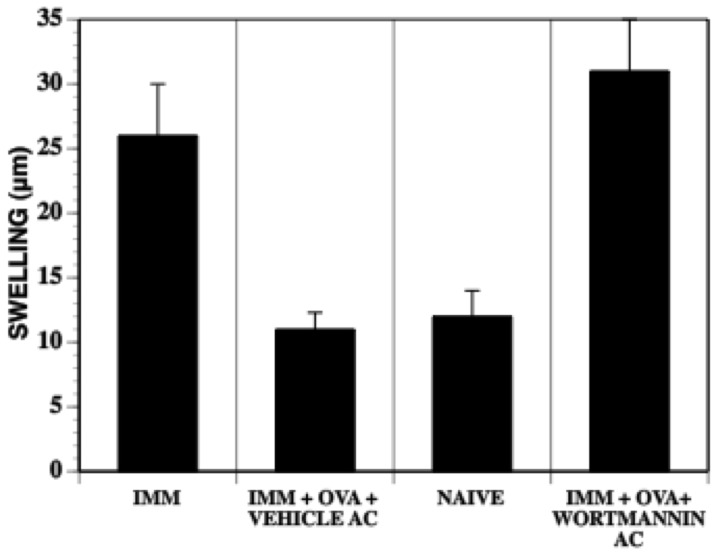
Intracameral injection of PI3 Kinase inhibitors along with the antigen prevent the development of ACAID. Mice received an intracameral injection of OVA or OVA +wortmannin. One week later the mice were immunized with OVA/CFA and one week later footpads were challenged with OVA. Footpad swelling was measured 24 hr after challenge and were compared between immunized mice, naïve mice and those that received an intracameral injection of vehicle + OVA with that of wortmannin and OVA. The experiments were repeated 3X.

Activation of the PI3 kinase pathway and the subsequent phosphorylation of Akt is known to play a role in activation state of monocytes [24]. Inhibition of this PI3 kinase/Akt pathway by wortmannin along with OVA antigen at the time of the intracameral injection prevented the induction of DTH suppression (Fig. 6). However, wortmannin treatment over a range of doses did not alter the levels of CCL-2 (data not shown). In summary, the suppression of DTH to OVA was not induced by the intracameral injection of OVA mice in receiving an intracameral injection of OVA and (i) a soluble receptor for TGF-β, (ii) an inhibitor selective for a TGF-β-associated kinase (Fig. 5), wortmannin or by LY294002, an alternative inhibitor of cytosolic PI3 kinase (Fig. 6, data not shown) even though intracameral injection- induced CCL2 levels in the aqueous humor were not affected in mice that received these treatments.

**Figure 7:**
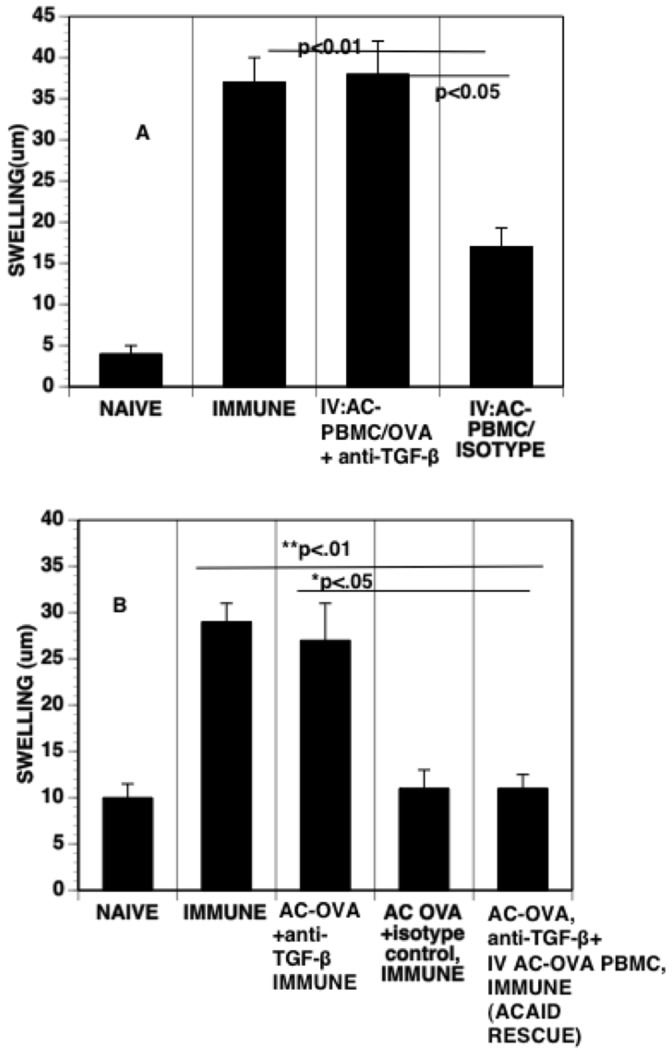
AC- PBMCs from Anti-TGF-β-treated animals do not transfer DTH suppression to OVA however intravenous injection of AC- PBMCs to those animals receiving anti-TGF-β+ OVA intracameral injections rescues the suppression of DTH. (A) Anti-TGF-β treatment at the time of intracameral injection of antigen blocks the ability of AC-PBMCs to induce the suppression of DTH. Twenty-four hr after intracameral injection of OVA and anti-TGF-β, PBMCs were recovered and 1×10^6^ recovered cells were injected IV into the naïve mice. These recipient mice were subsequently immunized with OVA/CFA. Seven days after immunizing, footpads of the mice were challenged subcutaneously and the relative increase in footpad swelling was compared between the immunized control mice and naïve mice. Each group in the final DTH measurement had 5 mice. The experiment was repeated 3X, (B) Animals receiving intracameral injections of anti-TGF-β +OVA were injected IV at 24 hours with AC-PBMCs recovered from animals receiving intracameral injections of OVA only. The recipient mice sere then immunized with OVA/CFA. Seven days after immunizing DTH measurements were done as described above. The experiment was repeated twice.

The adoptive transfer of immunocompetent cells from naïve or immunized experimental animals to other animals via intravenous injection is a long-established maneuver to investigate and isolate the function of the cells [1], [2], [5], [7], [11]. The induction of DTH is suppressed and antigen-specific regulatory T cells are induced in mice receiving an intravenous injection of PBMC recovered 24 hr after mice received an intracameral injection of antigen. Splenic antigen-specific CD8^+^ regulatory T cells are induced by the intravenous injection of mice with F4/80^+^ cells recovered from mice receiving an intracameral injection of antigen (reviewed in [1], [2]). Accordingly, we used this procedure to determine whether PBMC recovered from mice receiving OVA and anti-TGF-β antibodies transfer the suppression of DTH when injected intravenously into naive mice that are immunized subsequent to the injection of the AC-PBMC. Moreover, to ensure that the intracameral injection of anti-TGF-β antibodies did not affect cells in the thymus or spleen, mice receiving an intracameral injection of anti-TGF-β and OVA subsequently received intravenously PBMC recovered from donors that 24 hr previously had received an intracameral injection of OVA only. These recipients were then immunized to induce DTH to OVA. DTH was expressed in recipients that received PBMC recovered from mice that received an intracameral injection of OVA and anti-TGF-β (Fig. 7A) but was not expressed in the recipients of monocytes recovered from donors that received an intracameral injection of OVA and isotype control antibody (Fig. 7A). However, DTH to OVA was suppressed in mice that received an intracameral injection of anti-TGF-β and OVA and intravenous AC-PBMCs recovered from donors that received an intracameral injection of OVA only (Fig. 7B) (indicating that the intracameral injection of anti-TGF-β did not affect cells in the thymus or spleen. Therefore, the *in situ* neutralization of TGF-β or the signal pathway for TGF-β in the anterior chamber prevents the induction of PBMC that transmit ACAID.

## Discussion

The intracameral injection of antigen induces a CCR2-dependent infiltration of circulating cells into the anterior chamber that includes F4/80^+^ monocytic cells associated with the peripheral induction of splenic antigen-specific regulatory T cells that induce or effect the systemic suppression of DTH. Naïve F4/80^+^ monocytes associated with the iris and circulating F4/80^+^ monocytes from naïve or immunized mice do not induce antigen-specific regulatory T cells that suppress DTH [1], [2], [8]–[10]. Moreover, aqueous humor and antigen will induce such immunoregulatory monocytes *ex vivo* [9], [23]. In aggregate, our observations suggest that the transit of circulating monocytes through the anterior chamber in response to the intracameral injection is a an essential event that likely induces an immunoregulatory phenotype in the infiltrated cells which then recirculate after their drainage through aqueous humor into the venous circulation. Recently, we demonstrated that there is an infiltration of F4/80^+^ cells into the anterior chamber after an intracameral injection that is compromised in either CCL2 or CCR2-deficient mice [5]. These results suggested that the recruitment of the circulating monocytes to the anterior chamber is a response to the trauma of the injection as CCR2 is required for the recruitment of monocytes to other sites of injury [25]–[36]. The injection of PBS only or a simple “needle stick” induces a lesser infiltration of monocytes into the anterior chamber [5]. However, the lack of antigen in the intracameral injection does not induce immunoregulatory monocytes. Because cells recovered from the irides of mice receiving an intracameral injection of antigen or iris cells pulsed with antigen and TGF-β induce antigen-specific regulatory T cells, it is likely that the monocytes that infiltrated the anterior chamber after an intracameral injection of antigen gain antigen in the anterior chamber from resident dendritic cells in the iris and ciliary body that have taken up the antigen [10], [12], [13]. Surprisingly, the early events in the anterior chamber after an intracameral injection of antigen that recruit and induce an immunoregulatory phenotype in the infiltrated monocytes have not been detailed. Accordingly, we investigated further the early events that are reflected in aqueous humor after intracameral injection.

Three-6 hr after the intracameral injection of OVA there is a rapid rise in aqueous humor of the chemokines CCL2 and CCL7, the ligands for CCR2 [34]–[39]. There is a minimal rise in CCL2 and CCL7 in aqueous humor after a simple needle stick. Because an intracameral injection of OVA induces a greater rise in CCL2 and CCL7 than the insertion of a needle only or an intracameral injection of PBS only, these results suggest that the stronger the irritant, the greater the rise in CCL2. This may explain why the induction of ACAID to soluble bovine albumin required a larger bore needle for the intracameral injection than that required to induce ACAID to trinitrophenylated red blood cells [40]. The trinitrophenylated erythrocytes injected in that report [40] that were used to induce ACAID to trinitrophenol were likely more irritating than BSA. We have made similar observations when comparing trinitrophenylated albumin to OVA for the induction of ACAID (RE Cone, Yen Lemire, unpublished observations). It is notable that 16–24 hr after the intracameral injection the levels of CCL2 and CCL7 in aqueous humor decline significantly suggesting that the trauma of injection and the antigen is declining in this time period. This decline is consistent with a reduction in the infiltration of monocytes into the anterior chamber 8 hours after the intracameral injection of antigen [5].

Different cells associated with inflammation infiltrate the anterior chamber after an intracameral injection [5]. However, because only the F4/80^+^cells in the iris and the circulation after an intracameral injection of antigen induce ACAID, we directed our attention to the F4/80^+^ cells. There is a near total absence of the infiltration of F4/80^+^, CD11b^hi^, Gr1^hi^ cells into the anterior chamber in CCR2 or CCL2 -null mice that received an intracameral injection of antigen. Because CCR2 or CCL2 null mice do not produce ACAID-inducing circulatory monocytes [5], our observations suggest that these infiltrated F4/80^+^, Gr1^+^ (Ly6C^hi^) monocytes are the ACAID-inducing F4/80^+^ monocytes. Although it is well established that the ACAID-inducing (immunoregulatory) monocyte is F4/80^+^, our observations characterize these cells further to identify this important subset of cells. 100% of the F4/80^+^ cells that infiltrate the anterior chamber after intracameral injection express GR1 (fig. 2) and CD11b. We have observed that CD11b^+^ cells recovered from the iris after an intracameral injection of antigen induce immunoregulatory thymocytes essential for the induction of ACAID (R. Sharafieh and RE Cone, unpublished data). Therefore, the F4/80,GR1^+^ cells are likely immunoregulatory monocytes (after traversing the anterior chamber). Further phenotyping of these monocyte subsets demonstrated that they show the distinct pattern of freshly recruited hematopoetic cells (CD45^+^, Ly6C^hi^, 7/4 (Ly6B)^hi^, F4/80^+^, CD11b^hi^, CD115^+^, CD49B^+^ and CD62L^+^).Therefore, these monocytes have the characteristics of inflammatory monocytes [19]. Very few of these cells were observed to be recruited into the anterior chamber in CCR2^–/–^ and CCL-2^–/–^ mice after the intracameral injection of antigen. In addition, in our previous study total PBMCs isolated from the blood of CCL2^–/–^ mice receiving an intracameral injection of antigen did not have the capacity to induce ACAID when transferred into wildtype naïve animals whereas wildtype mice AC-PBMCs could transfer ACAID when transferred in a similar way. As F4/80^+^ cells found in the blood of mice receiving an intracameral injection of antigen induce antigen- specific CD8^+^ regulatory T cells, the regulatory effector cell in ACAID [1], [2], [7]–[9], collectively these results indicate that these Gr1 (Ly6C) hi, CD11b hi, F4/80^+^ cells derived from a CCL2-dependent infiltration of these F4/80^+^ monocytes into the anterior chamber are likely the cells capable of inducing antigen- specific CD8^+^ regulatory T cells in the spleen. Peritoneal exudate cells pulsed *in vitro* with antigen and TGF-β, have also been shown to generate antigen-specific regulatory CD8^+^ T cells. Since the infiltration of blood monocytes in the peritoneal exudate in response to an intraperitoneal injection of thioglycollate is dependent on CCL2 [32], [41] and as these cells express an inflammatory monocyte phenotype [18], [32], we contend that the injury- responsive infiltrated cells in the anterior chamber are the functional F4/80^+^ cells capable of inducing ACAID.

In addition to the appearance in aqueous humor of CCL2 and CCL7 after the intracameral injection of antigen, there is a concomitant increase in TNF-α as shown previously by Ferguson and colleagues [16], [40]. The chemokine CCL2 is detected in aqueous humor 3 hr after the intracameral injection of antigen and anti-TNF-α reduced CCL-2 levels detected 3 hr after the intracameral injection of anti-TNF-α and antigen. These observations are consistent with those demonstrating that TNF-α stimulates the production of CCL2 [39] and suggest that TNF-α exerts an influence on the early production of CCL2 in the anterior chamber in response to the intracameral injection of antigen.

In the context of immune modulation by tumors there is evidence for the role of a TNF-α and TGF-β-mediated loop involving infiltration and modulation of macrophages [42]. In our investigation, although the intracameral injection of anti-TGF-β attenuated the production of TNF-α, the production of CCL-2 was not affected. Blockade of TNF- α resulted in significant reduction of CCL2 at the earlier 3 hr time point post intracameral injection but not at later time points. As evidenced by the kinetics of the appearance of CCL2 (Fig. 1), continuous production of CCL2 and (in tandem) continuous infiltration of monocytes allows an analysis of the infiltration of cells into the anterior chamber 16 hr post intracameral injection. This is demonstrated further by the lack of infiltration of monocytes in CCL2–/– mice even though TGF-βis present in these mice. As the production of TNF-α is dependent on the presence of TGF-β and the blockade of TGF-β did not change CCL2 levels or the infiltration of monocytes in CCL2–/– mice in response to an intracameral injection, we contend that TNF-α does not influence the continuous infiltration of monocytes. Moreover, the likely presence of TNF-α in the aqueous humor of CCL2–/– and CCR2–/– mice receiving an intracameral injection of antigen does not recruit circulating monocytes [5]. This lack of an effect of anti-TNF-α on CCL2 production at later time points and the recruitment of monocytes into the anterior chamber when the production of TNF-α is blocked by the injection of anti-TGF-β suggests that although TGF-β may have a moderate regulatory control on CCL2 production as reported for corneal injury of the eye [43], [44], the role for TGF-β and TNF-α in ACAID is not an influence on the infiltration of inflammatory monocytes into the anterior chamber. Moreover, the expression of TNF-α declines rapidly after 6 hours whereas the level of CCL2 is maintained well beyond 16 hours. Monocytes derived from blood recovered from CCL2 null mice that received an intracameral injection of antigen do not have the capacity to induce ACAID [5] and there is lack of significant infiltration of F4/80^+^ monocytes into the anterior chamber in response to an intracameral injection in CCL2^–/–^ mice (Fig. 2,3). As neutralization of TGF-β at the time of intracameral injection prevented the development of ACAID (Fig. 6), we investigated whether TGF-β blockade affected CCL2 levels in aqueous humor as well as the infiltration of these monocytes into the anterior chamber after an intracameral injection.

We did not observe any change in the infiltration of monocytes induced by an intracameral injection of antigen and anti-TGF -β. Similarly, TGF-β blockade via antibody, receptor kinase inhibitor or via soluble TGF-β receptor, did not affect the production of CCL2 (Fig. 4). These results indicate that TGF-β (and TNF-α) are not necessary for the sustained production of CCL2 induced by an intracameral injection or the consequent infiltration of monocytes into the anterior chamber. As TNF-α is known to upregulate the expression of cell adhesion molecules [45] and thus facilitate the extravasation of leucocytes, we determined the upregulation of ICAM-1 (the integrin which interacts with CD11b) [46] using confocal imaging. We did not find any change in the expression of ICAM-1 on the iris vasculature after AC injection (data not shown). TNF-α is only expressed transiently in aqueous humor after an intracameral injection and disappears by 12 hours. On the other hand, the presence of CCL2 (as well as CCL7) as well as the infiltration of cells lasts much longer. It is conceivable that there is continuous infiltration and egress of monocytes into and from the anterior chamber to venous circulation beyond the 12 hr time point. Since blockade of TNF-α at the time of intracameral injection has previously been reported to block the development of ACAID, it is likely that TNF–α plays a modulatory role in the generation of tolerogenic monocytes along with TGF-β. Although TNF-α is predominantly considered as an inflammatory and tissue -damaging cytokine there is evidence for its role in homeostatic, tissue healing [47], [48], or as an immunoregulatory cytokine [49]–[52].

In addition to the infiltration of monocytes, neutrophils (CD11b^hi^, Gr1^hi^ (Ly6G^hi^) but F4/80^−^ also infiltrate the anterior chamber after the intracameral injection. Typically, granulocytes have a higher side scatter and a lesser forward scatter when compared to monocytes. Taken together, the increase in aqueous humor of CCL2, CCL-7 and TNF-α indicates a mild, transient proinflammatory response in the anterior chamber that is induced by the intracameral injection. As previous studies have clearly implicated F480^+^ cells as the cells responsible for inducing ACAID, here we show that these cells infiltrate into the eye in response to CCL2. The infiltration of neutrophils in response to AC injection strengthens our assertion that it is an injury response to the injection. It is likely that induction of neutrophil-specific chemokines are responsible for their infiltration. At this juncture it is not clear if neutrophils have a role to play in the generation of immunoregulatory monocytes and therefore ACAID.

Neutralization of TGF-β by the intracameral injection of antibodies to TGF-β prevented the induction of the suppression of DTH consistent with previous reports showing that the capability of monocytes that have been treated *in vitro* with aqueous humor and antigen to induce suppression of DTH is dependent on TGF-β [1], [23]. Finally, a soluble receptor for TGF-β an inhibitor of a TGF-β-associated kinase, also prevented the induction of the suppression of DTH suggesting that the signaling pathway associated with the receptor for TGF-β is essential to activate the monocytes in the anterior chamber. The intracameral injection of antibodies to TGF-β does not inhibit the infiltration of monocytes suggesting that TGF-β does not recruit monocytes to the anterior chamber but does participate in the induction of a immunosuppressive phenotype in these cells. Importantly, monocytes recovered from mice receiving an intracameral injection of antigen (only) “rescued” the induction of ACAID when administered to mice receiving an intracameral injection of antigen and anti-TGF-β antibodies. This control ensured that the antibodies were not suppressing the systemic induction of regulatory T cells in the thymus and spleen.

In addition to blocking TGF-β with antibodies, the inhibition of TGF-β signaling was also effective in preventing the induction of ACAID. The activation of the PI3 kinase pathway has also been reported to promote tolerant monocytes [24]. Injection of wortmannin, an inhibitor of PI3 kinase pathway along with the antigen during the AC injection inhibited the development of the suppression of DTH after the intracameral injection of antigen. We then reasoned that perhaps this may be due to inhibition of CCL2 synthesis as was reported for CCL2 production by retinal pigment epithelial cells [53]. However, wortmannin injected over a range of doses did not inhibit the synthesis of CCL2 (data not shown) thus ruling out the involvement of PI3 kinase pathway in the AC injection induced chemokine synthesis.

Blockade of both TGF-β as well as PI3 kinase prevents the development of ACAID without altering the levels of CCL2 production. Accordingly, perhaps the PI3 kinase/Akt pathway may participate in the downstream signaling of TGF-β and thus take part in the induction of ACAID- inducing monocytes since TGF-β is known to engage non SMAD pathways including PI3kinase pathways [54], [55]. A recent report [56] demonstrated that tumor-derived TGF-β induced expression of microRNA-494 and the consequent activation of the Akt pathway in CD11b,Gr1^+^ myeloid-derived suppressor cells (MDSCs) was required for their accumulation as well as subsequent production of factors responsible for the facilitation of tumor spread. MDScs represent a heterogeneous group of cells including F4/80^+^Ly6C^hi^ monocytes. A recent report by Augier *et al* [57] suggests that that MDSCs are not a separate lineage but rather become suppressive after infiltration into the tumor in response to tumor-derived CCL2. It is notable that the myeloid-derived suppressor cells in that report had a phenotype similar to the F4/80,CD11b, GR1^+^ that infiltrated the anterior chamber after an intracameral injection of antigen and likely are the circulating cells that induce splenic regulatory T cells that effect ACAID. It is conceivable that these bone marrow-derived monocytes can behave as suppressor cells or inflammatory cells based on environmental cues. However, it is important to note that wortmannin treatment and the consequent blockade of PI3 kinase may affect additional targets that also block the development of ACAID. The PI3 kinase pathway is also critical to the downstream signaling of CD47, a receptor for thrombospondin, also instrumental in the induction of ACAID [58]–[62]. PI3 kinase is also known to be involved in signaling downstream of the CCR2 receptor [61], [62] and therefore may be important in MCP-1-mediated chemotaxis of monocytes into the anterior chamber.

CCL-2 and CCL-7 and TNF-α may also influence the induction of an immunosuppressive phenotype on the infiltrated monocytes. Our observations therefore confirm a previous report that TNF-α is required for the induction of ACAID [16]. Although we observed that this cytokine may promote the early production of CCL-2 in aqueous humor after an intracameral injection and may participate in the early recruitment of inflammatory monocytes to the anterior chamber, it is not responsible for the continuous recruitment of inflammatory monocytes into the anterior chamber. TNF-α is transiently expressed and the induction of CCL2 and the concomitant infiltration of inflammatory monocytes follows a much longer kinetics. Accordingly, TNF-α may play an immunoregulatory role. Taken together, our observations suggest that the initiation of ACAID in the anterior chamber may be factored into four stages: (i) A production of TNF-α and CCL2/CCL7 in aqueous humor, (ii) a CCL2-dependent recruitment of inflammatory monocytes into the anterior chamber after an intracameral injection, (iii) a TGF-β, TNF-α-dependent induction of a suppressive phenotype in the recruited monocytes, (iiii) an egress and recirculation of the recruited monocytes to the thymus and spleen where the participate in the induction of antigen-specific regulatory T cells.

Because circulating F4/80^+^ monocytes from naïve animals do not induce ACAID [5], [10], they must gain immunosuppressive abilities when they traverse the anterior chamber and gain the injected antigen. Quantitatively, F4/80^+^ peritoneal exudate cells treated with TGF-β and antigen *in vitro* [1], [6], [63], or F4/80^+^ monocytes recovered from the irides of mice that received an intracameral injection of antigen are more effective in inducing ACAID than circulating monocytes recovered from mice that received an intracameral injection of antigen (RE Cone, Y Lemire, unpublished observation) suggesting that the exposure of the monocytes to all of these inflammatory cytokines enriches for the ACAID-inducing monocyte.

Our results suggest that a moderate, proinflammatory response in the anterior chamber may induce a suppression of adaptive immune mechanisms to defend against the invader so that rather than protecting from future infection, ACAID may also increase susceptibility to a future infection. TGF-β *in situ* is essential for the induction of ACAID in part through an influence on the production of TNF-α and a likely influence on the induction of an immunosuppressive phenotype in the recruited monocytes. These results are in agreement with a recent report showing the lack of TGF-β in aqueous humor after cervical ganglion ablation as the most likely cause of the blockade of ACAID [64] by cervical ganglionectomy. Further evidence for the inflammatory nature of the recruited monocytes is suggested by pulsing peritoneal exudate cells with antigen and TGF-β *in *vitro or in the presence of aqueous humor to generate immunoregulatory monocytes (reviewed in [1]). The infiltration of monocytes into the peritoneal cavity induced by thioglycollate is dependent on CCL2 [32], [41], [65]. Accordingly, these cells express CCR2.

While the CCR2 subset of monocytes are cells that have undergone recent myelopoiesis and which still express 2Gr1 (Ly6C) are recruited into sites of inflammation, they are also known to participate in diverse functions like tissue healing [36] atherogenesis [66], nephropathy [67], tumor metastasis, angiogenesis [68] and fibrosis [26]. Monocytes may acquire antigen from other cells and present them to T cells in the periphery [69]. Although there is no consensus as to whether there are separate sublineages within this (CCR2) subset or if these cells are influenced by microenvironmental cues and thus are capable of plasticity[70]–[72], there is growing appreciation of their plasticity to be able to carry out diverse immunological or non- immunological functions. Moreover, the importance of the CCL2/CCR2 axis in recruiting inflammatory monocytes to sites that may activate a regulatory phenotype in these cells indicates the importance of polymorphisms in this axis that could impact on susceptibility to autoimmune disease [73], [74]. Accordingly, ACAID demonstrates an influence on circulating inflammatory monocytes by the environment surrounding the monocytes.
